# Editing of the Proteolytic System of Lactococcus lactis Increases Its Bioactive Potential

**DOI:** 10.1128/AEM.01319-20

**Published:** 2020-09-01

**Authors:** Chenxi Huang, Jan Kok

**Affiliations:** a Department of Molecular Genetics, Groningen Biomolecular Sciences and Biotechnology Institute, University of Groningen, Groningen, The Netherlands; University of Buenos Aires

**Keywords:** bioactive peptides, intracellular peptidomics, *Lactococcus lactis*, proteolytic system

## Abstract

Large-scale mass spectrometry-based peptidomics for bioactive-peptide discovery is relatively unexplored because of challenges in intracellular peptide extraction and small-peptide identification. Here, we present an analytical pipeline for large-scale intracellular peptidomics of Lactococcus lactis. It entails an optimized sample preparation protocol for L. lactis, used as an “enzyme complex” to digest β-casein, an extraction method for its intracellular peptidome, and a peptidomics data analysis and visualization procedure. In addition, we proofread the publicly available bioactive-peptide databases and obtained an optimized database of bioactive peptides derivable from bovine β-casein. We used the pipeline to examine cultures of L. lactis MG1363 and a set of 6 isogenic multiple peptidase mutants incubated with β-casein. We observed a clearly strain-dependent accumulation of peptides with several bioactivities, such as angiotensin-converting enzyme (ACE)-inhibitory, dipeptidyl peptidase 4 (DPP-IV)-inhibitory, and immunoregulatory functions. The results suggest that both the number of different bioactive peptides and the bioactivity diversity can be increased by editing the proteolytic system of L. lactis. This comprehensive pipeline offers a model for discovery of bioactive peptides in combination with other proteins and might be applicable to other bacteria.

**IMPORTANCE** Lactic acid bacteria (LAB) are very important for the production of safe and healthy human and animal fermented foods and feed and, increasingly more, in the functional food industry. The intracellular peptidomes of LAB are promising reservoirs of bioactive peptides. We show here that targeted genetic engineering of the peptide degradation pathway allows steering the composition of the peptide pool of the LAB Lactococcus lactis and production of peptides with interesting bioactivities. Our work could be used as a guideline for modifying proteolytic systems in other LAB to further explore their potential as cell peptide factories.

## Abstract (precis)

Lactic acid bacteria (LAB) are very important for the production of safe and healthy human and animal fermented foods and feed and, increasingly more, in the functional food industry. The intracellular peptidomes of LAB are promising reservoirs of bioactive peptides. We show here that targeted genetic engineering of the peptide degradation pathway allows steering the composition of the peptide pool of the LAB Lactococcus lactis and production of peptides with interesting bioactivities. Our work could be used as a guideline for modifying proteolytic systems in other LAB to further explore their potential as cell peptide factories.

## INTRODUCTION

Members of the diverse group of lactic acid bacteria (LAB) have been associated with food fermentations since ancient times. LAB were used as starter cultures for dairy production more than 100 years ago, which has given them their current industrial and economic importance (1). However, the value of those fermented products, such as yogurt or cheese, has remained restricted to providing basic nutrition, flavor, and texture. The potential of LAB as production organisms for functional foods is still largely unexplored. Lactococcus lactis, for instance, depends on the milk protein casein as the major source of nitrogen, since it is auxotrophic for several amino acids. Casein degradation is accomplished in a three-step process. First, the extracellular cell envelope-associated proteinase (PrtP) degrades casein into oligopeptides. Second, (a selection of) these peptides are internalized via an oligopeptide transport system (Opp). In the third and last step, multiple peptidases hydrolyze the oligopeptides into smaller peptides and, ultimately, into amino acids that are then available for *de novo* protein synthesis and other metabolic activities (2).

Functional food for health promotion or disease risk reduction has attracted the interest of food industries worldwide, with dairy products as one of the most popular categories. Milk proteins encode bioactive-peptide sequences, which can be released by hydrolysis (3). These short peptides can display a spectrum of biological functions, such as angiotensin-converting enzyme (ACE)-inhibitory, dipeptidyl peptidase 4-inhibitory (DPP-IV-I), immunoregulatory, antioxidant, antimicrobial, and opioid activities. Bioactive peptides from milk proteins can be obtained via the action of microbial or nonmicrobial enzymes. Milk fermentation processes executed by LAB are preferable ways to release these peptides because of the food grade safety status of these organisms. Moreover, proteolytic systems of LAB, especially that of L. lactis, have been comprehensively studied with respect to the genes and enzymes involved and their regulation (4, 5).

A great deal of research has focused on the production of milk-derived bioactive peptides using LAB. Two major ways of bioactive-peptide discovery can be discerned. First, casein proteins are either digested by a purified digestive enzyme (trypsin) or LAB proteinase(s), after which the obtained products are identified (6–8). Second, an LAB cell culture is mixed with milk proteins, and the supernatant is subsequently further characterized (9–11). These studies have identified numerous casein-derived bioactive peptides, most of which having ACE-inhibitory activity. From an application point of view, the costs of employing purified enzymes are too high for industrial-scale use. On the other hand, only utilizing the culture supernatant of proteolytically active cells does not exploit the full potential of the LAB, as in that case only the proteinase specificity is being utilized while the activities of the more-than-10 intracellular peptidases and possible hidden intracellular bioactive peptides are being ignored.

Our understanding of the intracellular peptide pool in LAB during growth in a milk medium and the possible presence of bioactive variants is limited to nearly absent due to the technical obstacles of preparing and separating the complex samples and the subsequent identification of the small peptides (12). Recent rapid developments in the technology of nanoscale liquid chromatography coupled to tandem mass spectrometry (nanoLC-MS/MS) and in algorithms for peptide identification have resulted in a dramatic increase in research in proteomics and its subfield peptidomics (13, 14).

In this study, we engineered the proteolytic system of the L. lactis model strain MG1363 and describe a robust and comprehensive analytical framework of cell-casein incubation conditions, intracellular peptidome extraction, data analysis and visualization, and, ultimately, identification of casein-derived bioactive peptides produced by L. lactis MG1363 and six of its isogenic peptidase mutants. As proof of concept, this work offers a pipeline for the analysis and visualization of the intracellular peptidome of bacteria and explores the possibility of applying L. lactis (or other bacteria) as a cell factory to produce bioactive peptides.

## RESULTS

### Engineering of an L. lactis proteolytic system.

The aim of this study was to build an analytical framework for the analysis of the intracellular peptidome of L. lactis and to discover (putative) bioactive peptides obtained upon degradation of β-casein by the organism. To kick-start β-casein degradation by the L. lactis model strain MG1363, an extracellular cell wall-anchored proteinase, PrtP (caseinase), is needed. The parent strain of L. lactis MG1363, L. lactis NCDO712, carries the 55-kb PrtP proteinase and lactose plasmid pLP712. This plasmid is too large to easily be reintroduced in MG1363 and its peptidase knockout derivatives, while it also contains one of the oligopeptidase genes, *pepF_1_* (15). Therefore, a new plasmid that encodes the proteinase PrtP and its maturase PrtM (16) from pLP712 was constructed and named pCH020. L. lactis MG1363 possesses 15 intracellular peptidases that together degrade the PrtP-liberated casein-derived oligopeptides that are internalized by the oligopeptide permease Opp. The peptidase complement will ultimately result in the decomposition of the oligopeptides into shorter peptides and free amino acids. Undigested oligopeptides and peptidase-digested shorter versions of these peptides might possess bioactivities.

**FIG 1:**
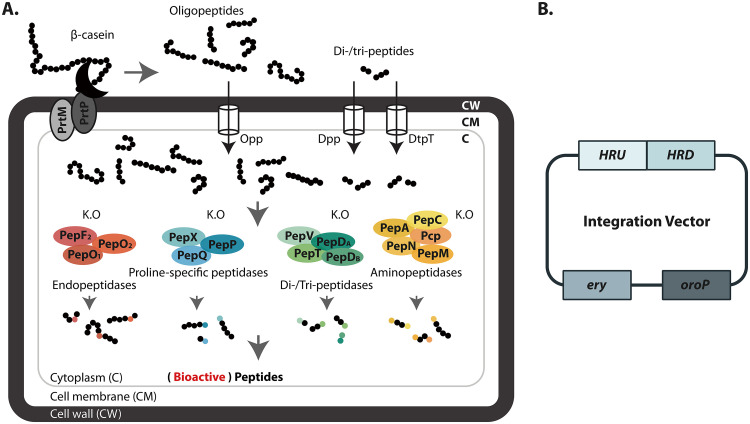
Peptidomics and bioactive-peptide discovery in L. lactis. (A) Schematic representation of the mutated proteolytic system of L. lactis MG1363 with the cell envelope-associated proteinase (PrtP). β-Casein hydrolysis is initiated by PrtP after its autoproteolytic activation with the aid of PrtM (16). Subsequently, the oligopeptides are transported into the cells by the Opp transport system, while di-/tripeptides are internalized by the Dpp or DtpT transport system. The peptides are then degraded by the concerted action of 15 peptidases, which are classified and colored by their indicated cleavage specificity. When a specific combination of peptidase genes is deleted, certain peptides will stay intact. Theoretically, small (bioactive) peptides, instead of free amino acids, will accumulate intracellularly. (B) General sketch of the peptidase gene knockout vector. The integration vector is based on plasmid pCS1966 (17). It contains an erythromycin resistance gene (*ery*), an orotate transporter gene (*oroP*), and a knockout cassette carrying two homology regions, one containing the region upstream of the gene to be deleted (HRU) and the other encompassing a region downstream thereof (HRD), for double crossover integration at a certain peptidase gene locus.

By removing different (groups of) peptidases, more and a greater variety of intracellular peptides should accumulate, increasing the chance of discovering (novel) bioactive peptides (Fig. 1A). A total of 37 single and multiple isogenic peptidase mutants were constructed from L. lactis MG1363 (Table 1) by employing 16 peptidase gene replacement vectors based on the replication-deficient plasmid pCS1966 (17) (Fig. 1B). Several multipeptidase deletion mutants were designed based on peptidase functional groups. Thus, four mutants were obtained in which all endopeptidases (MGΔ*pepOF_2_O_2_*), all aminopeptidases except PepM (MGΔ*pepANCpcp*; see below), all proline-specific peptidases (MGΔ*pepXPQ*) or the di-/tripeptidases (MGΔ*pepVD_A_TD_B_*) had been removed. In addition, in a strategy to delete as many peptidase genes as possible, half of them were deleted in strains MGΔ*pepNXOTCF_2_O_2_* and MGΔ*pepNXOTCVD_A_*. Note that *pepM* is an essential gene in MG1363 and is thus present in all peptidase mutants.

**TABLE 1:** Strains used in the peptidomic experiment

Strain	Species	Description	Short name	Antibiotic resistance	Reference
MG1363	L. lactis	Plasmid-free derivative of NCDO712	MG1363		53
CH000	L. lactis	MG1363 with plasmid pCH020	MG1363(PrtP^+^)	Ery^*a*^	This study
IM14	L. lactis	MG1363 Δ*pepNXOTC*	MGΔ*pepNXOTC*		54
CH001	L. lactis	MG1363 Δ*pepF_2_*	MGΔ*pepF_2_*		This study
CH002	L. lactis	MG1363 Δ*pepO_2_*	MGΔ*pepO_2_*		This study
CH003	L. lactis	MG1363 Δ*pepA*	MGΔ*pepA*		This study
CH004	L. lactis	MG1363 Δ*pepP*	MGΔ*pepP*		This study
CH005	L. lactis	MG1363 Δ*pepV*	MGΔ*pepV*		This study
CH007	L. lactis	MG1363 Δ*pcp*	MGΔ*pcp*		This study
CH008	L. lactis	MG1363 Δ*pepQ*	MGΔ*pepQ*		This study
CH009	L. lactis	MG1363 Δ*pepD_A_*	MGΔ*pepD_A_*		This study
CH010	L. lactis	MG1363(pLP712Δ*pepF_1_*)	MGΔ*pepF_1_*		This study
CH011	L. lactis	MG1363 Δ*pepO*	MGΔ*pepO*		This study
CH012	L. lactis	MG1363 Δ*pepC*	MGΔ*pepC*		This study
CH013	L. lactis	MG1363 Δ*pepN*	MGΔ*pepN*		This study
CH014	L. lactis	MG1363 Δ*pepX*	MGΔ*pepX*		This study
CH015	L. lactis	MG1363 Δ*pepT*	MGΔ*pepT*		This study
CH016	L. lactis	MG1363 Δ*pepDB*	MGΔ*pepD_B_*		This study
CH017	L. lactis	MG1363 Δ*pepOF_2_*	MGΔ*pepOF_2_*		This study
CH018	L. lactis	MG1363 Δ*pepOF_2_O_2_*	MGΔ*pepOF_2_O_2_*		This study
CH019	L. lactis	MG1363 Δ*pepVD_A_*	MGΔ*pepVD_A_*		This study
CH020	L. lactis	MG1363 Δ*pepVD_A_T*	MGΔ*pepVD_A_T*		This study
CH021	L. lactis	MG1363 Δ*pepVD_A_TD_B_*	MGΔ*pepVD_A_TD_B_*		This study
CH022	L. lactis	MG1363 Δ*pepAN*	MGΔ*pepAN*		This study
CH023	L. lactis	MG1363 Δ*pepANC*	MGΔ*pepANC*		This study
CH024	L. lactis	MG1363 Δ*pepANCpcp*	MGΔ*pepANCpcp*		This study
CH025	L. lactis	MG1363 Δ*pepXP*	MGΔ*pepXP*		This study
CH026	L. lactis	MG1363 Δ*pepXPQ*	MGΔ*pepXPQ*		This study
CH027	L. lactis	MG1363 Δ*pepNXOTCF_2_*	MGΔ*pepNXOTCF_2_*		This study
CH028	L. lactis	MG1363 Δ*pepNXOTCF_2_O_2_*	MGΔ*pepNXOTCF_2_O_2_*		This study
CH029	L. lactis	MG1363 Δ*pepNXOTCV*	MGΔ*pepNXOTCV*		This study
CH030	L. lactis	MG1363 Δ*pepNXOTCVD_A_*	MGΔ*pepNXOTCVD_A_*		This study
CH031	L. lactis	CH020 with plasmid pCH018	MGΔ*pepOF_2_O_2_ (*PrtP^+^)	Ery	This study
CH032	L. lactis	CH021 with plasmid pCH020	MGΔ*pepVD_A_TD_B_* (PrtP^+^)	Ery	This study
CH033	L. lactis	CH024 with plasmid pCH020	MGΔ*pepANCpcp* (PrtP^+^)	Ery	This study
CH034	L. lactis	CH026 with plasmid pCH020	MGΔ*pepXPQ* (PrtP^+^)	Ery	This study
CH035	L. lactis	CH028 with plasmid pCH020	MGΔ*pepNXOTCF_2_O_2_ (*PrtP^+^)	Ery	This study
CH036	L. lactis	CH030 with plasmid pCH020	MGΔ*pepNXOTCVD_A_* (PrtP^+^)	Ery	This study
DH5α	E. coli	*fhuA2 lac*Δ*U169 phoA glnV44* ϕ80' *lacZ*ΔM15 *gyrA96 recA1 relA1 endA1 thi-1 hsdR17*			55

a Ery, erythromycin.

Each peptidase mutant that was examined with respect to its peptidome carried the plasmid pCH020. The strains carrying pCH020 were labeled PrtP^+^, e.g., MG1363(PrtP^+^) (Table 1), but in the presentation of the results below, the addition PrtP^+^ is omitted for reasons of simplicity.

### Optimization of the intracellular peptidomics workflow.

**FIG 2:**
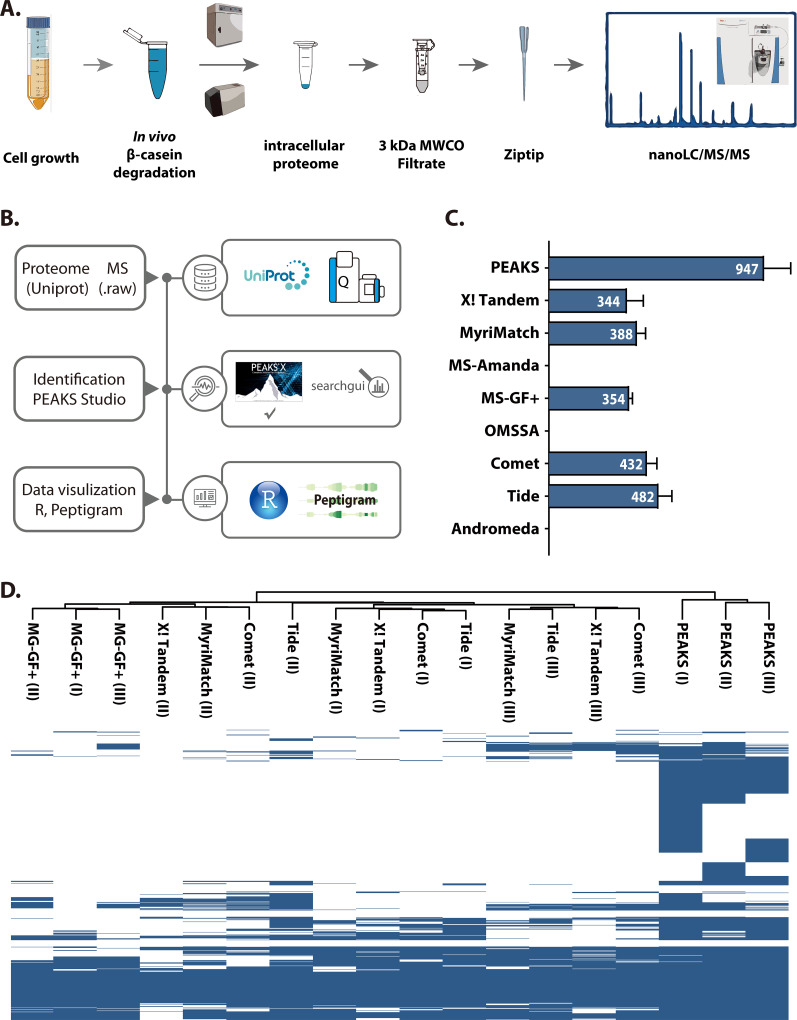
L. lactis intracellular peptidomic sample preparation and data analysis optimization. (A) Sample preparation workflow (for details, see Materials and Methods). Cells from exponentially growing L. lactis MG1363 and its peptidase knockout mutants were incubated under slow rotation (180 rpm) in a β-casein solution (4 mg/ml) for 3.5 h, after which they were disrupted by mini-beadbeating. The intracellular proteome was extracted and passed through a 3-kDa-molecular-weight-cutoff filter. The filtrate, which is the intracellular peptidome, was desalted using C_18_ Zip-tips and analyzed by nanoLC-MS/MS. (B) Computational data analysis. Mass spectrum data [MS(.raw)] output and the proteome of the sequence of L. lactis MG1363 plus β-casein (both obtained from UniProt) were used as inputs for PEAKS studio analysis. The output from PEAKS was further visualized through R programming and the peptidomics visualization web server Peptigram. (C) Comparison of 9 commonly used search engines on the L. lactis MG1363 peptidome. The bar chart shows the number of unique peptides identified by each search engine. The number (white) is the mean value from biological triplicates. Standard deviation is shown for each bar. (D) Map of the presence/absence of unique peptides from panel C, identified by the indicated 6 search engines on the biological replicates (roman numerals).

In order to obtain high-quality LC-MS data and convincing peptide identification results, three aspects were considered: the quality of *in vivo* β-casein degradation, L. lactis intracellular peptidome extraction, and the peptide identification algorithm. To optimize sample preparation for LC-MS-based intracellular peptidomics, each step of the workflow was considered (Fig. 2). Our previous time series transcriptome sequencing (RNA-seq) results (18) revealed that the proteolytic system of L. lactis MG1363 is relatively highly active during the log phase of growth, and thus we chose to harvest cells in the mid-log phase (optical density at 600 nm [OD_600_] ≈ 1) to start the *in vivo* β-casein degradation. Preliminary experiments employing different β-casein concentrations (1, 2, or 4 mg/ml) and incubation times (0.5, 1, 2, or 4 h or overnight) were tested, and ultimately 4 mg/ml β-casein and 3.5 h of incubation time were chosen to achieve a proper balance between sample quality and time management (data not shown). Since Gram-positive bacteria such as L. lactis have a thick cell wall, obtaining the intracellular peptidome requires cell disruption using mechanical forces (19). Ultrasonication and the mini-beadbeater were tested, with both setups yielding similar results. However, when performing nanoLC-MS on the samples, it was observed that the sonicator probe introduced an overwhelming polyethylene glycol (PEG) contamination in the peptide fraction between 150 and 600 Da. This problem did not occur using glass beads and the mini-beadbeater to break the cells (data not shown). Since the focus is to identify bioactive peptides, the intracellular proteome was enriched for small peptides by using the flowthrough obtained after centrifuging the proteome sample over a 3-kDa-cutoff filter prior to analysis by nanoLC-MS/MS (see Materials and Methods). All peptidome samples obtained in this way were analyzed in biological triplicates. Excluding the β-casein *in vivo* degradation time, this optimized sample preparation protocol for rapid intracellular peptide extraction, from the breaking open of the cells to the filtering through the 3-kDa-cutoff filter, can be performed within 1 h.

After obtaining the peptidome raw data, in order to find the most suitable search algorithm for our data set, we tested 9 commonly used search engines for peptide identification. The PEAKS search engine (20) was used in combination with the commercial proteomics platform PEAKS studio, while for the other 8, SearchGUI, an open-source interface configuring and running proteomics searches (21), was employed. All search engines were tested under the same setting using the raw data from MG1363 triplicates (see details in Materials and Methods). As Fig. 2C shows, 5 out of 8 search engines in SearchGUI gave approximately the same level of unique peptide identifications: Tide (22) and Comet (23) identified more peptides (both >400) than X! Tandem (24), MyriMatch (25), and MS-GF+ (26) (all three >300), while MS Amanda (27), OMSSA (28), and Andromeda (29) did not work properly for our data sets. Despite the fact that the MS-GF+ identification output is lower (354), it has good reproducibility since the triplicates examined with MS-GF+ are clustered in the heat map shown in Fig. 2D. PEAKS identified significantly more unique peptides (947) than the other search engines and was also reproducible (Fig. 2D). Thus, we chose PEAKS as the search engine in this work. The identified peptides and proteins were further analyzed and visualized with R and the web tool Peptigram (30).

### Peptidase deletion results in different intracellular peptidomes.

**FIG 3:**
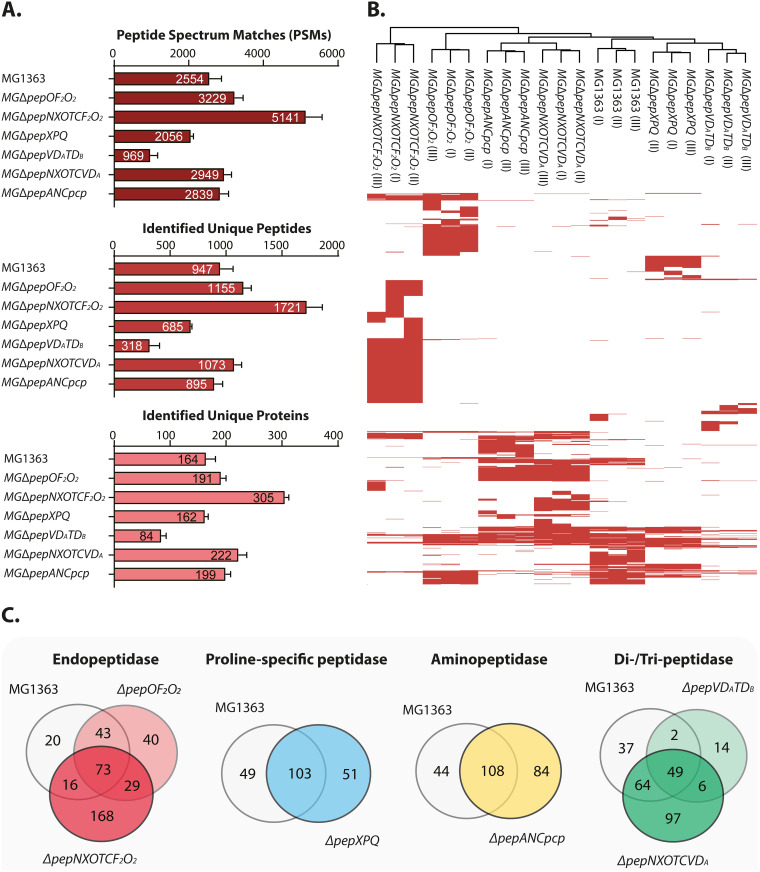
Overview of mass spectrum results from PEAKS studio of intracellular peptidomes of L. lactis MG1363 and 6 of its peptidase mutants. (A) Peptide spectrum match (PSM)-identified unique peptides and proteins identified in the peptidomes of the indicated strains. The bar charts show the mean value, the exact value of which is shown in the bar, and standard deviation from biological triplicates of each strain. (B) Map of the presence/absence of identified unique peptides from panel A. (C) Venn diagrams of identified unique proteins from panel A (http://bioinformatics.psb.ugent.be/webtools/Venn/).

**FIG 4:**
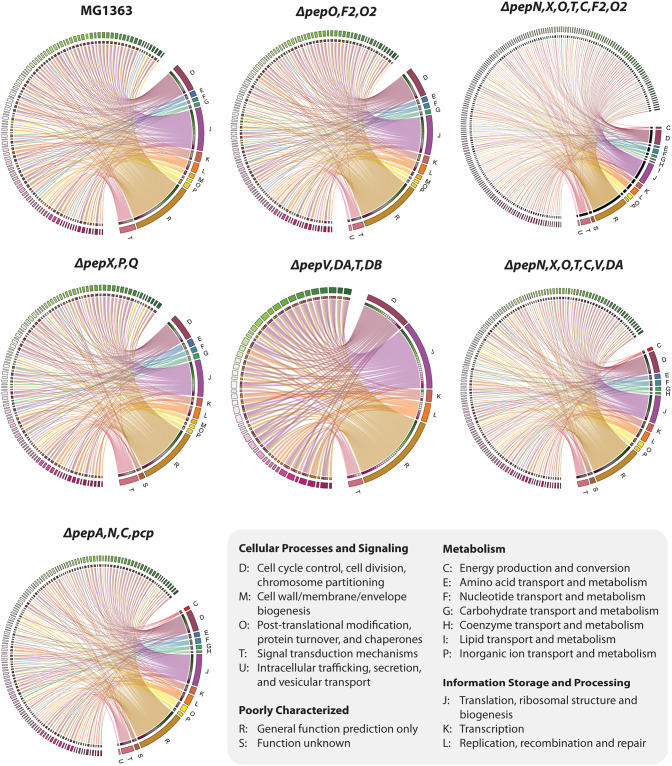
Ontology analysis of unique proteins identified in the intracellular peptidomes of L. lactis MG1363 and the 6 indicated isogenic *pep* mutants. Correlation of each protein (outer circle on the left side of each graph) and Clusters of Orthologous Groups (COG) category is represented by lines. Abbreviations of COGs are listed in the inset. The figure was generated by R package Circlize (50).

Figures 3 and 4 give a more detailed account of the results obtained for the 7 strains tested, ranging from their peptidomics profiles to gene ontology analyses. Figure 3A shows the numbers of identified peptide spectrum matches (PSMs), of unique peptides, and of unique proteins in the various intracellular peptidomes. From this figure it is clear that the deletion of all endopeptidase genes significantly increases the intracellular peptide pool. For strain MGΔ*pepOF_2_O_2_*, 3,200 PSMs were detected, while 2,600 PSMs were obtained from the sample of the parent strain MG1363. When in the strain lacking all endopeptidase activity, peptidases from other functional groups, namely, the aminopeptidases N and C, the proline-specific peptidase PepX, and the tripeptidase PepT were removed (MGΔ*pepNXOTCF_2_O_2_*), the number of PSMs almost doubled compared to that in MG1363 (5,100 versus 2,600) (Fig. 3A). Also, a higher number of PSMs (2,900) was detected in strain MGΔ*pepNXOTCVD_A_* than in MG1363, but it was much lower than in MGΔ*pepNXOTCF_2_O_2_*. As these two multipeptidase mutant strains have 5 peptidase gene deletions in common, it is highly likely that deletion of the endopeptidases PepF_2_ and PepO_2_ is responsible for most of the increase in PSMs. Slightly more PSMs were detected in the multiaminopeptidase mutant strain MGΔ*pepANCpcp* than in MG1363. All these observations show that by eliminating (multiple) general peptidases, the intracellular peptide pool will increase in both quantity and diversity. When peptidases with similar specificities are removed, such as in strain MGΔ*pepXPQ*, lacking several proline-specific peptidases, or in a strain deficient for peptidases playing important roles in the last stages of peptide degradation (the di-/tripeptidase mutant MGΔ*pepVD_A_TD_B_*), a dramatic decrease in the number of unique identified peptides is seen relative to that in strain MG1363. Strain MGΔ*pepVD_A_TD_B_* produced less than half the PSMs of MG1363, which might be due to the fact that deletion of *pepV* affects cell wall synthesis, which ultimately disturbs other biological processes such as nitrogen metabolism (31).

Hierarchical clustering of the identified peptides in the triplicate samples of each strain was performed to assess the robustness of the developed methodology. The results presented in Fig. 3B show that the biological replicates of each strain delivered data of good quality and reproducibility. The proteins identified in each mutant were compared with those of MG1363 on the basis of their functional grouping (Fig. 3C). Approximately two-thirds of the proteins identified in the peptidome of L. lactis MG1363 can be detected in each of the peptidase mutants. Notably, MGΔ*pepNXOTCF_2_O_2_* and MGΔ*pepNXOTCVD_A_* are the top two strains with respect to the number of unique proteins (168 and 97, respectively). Gene ontology (GO) enrichment analyses were performed in order to investigate the functional profiles of the identified proteins from each strain and to evaluate the effects of peptidase deletions on the peptidomes of the respective *pep* mutants. The cellular function grouping of the identified proteins of MG1363 and its six isogenic *pep* mutants is shown in Fig. 4. For well-characterized proteins, the top three significantly enriched categories in all strains are translation (J), cell cycle control (D), and replication (J). Around one-quarter to one-third of the proteins are poorly characterized (R and S). Figure S1 in the supplemental material shows the details of the overlap in the peptidomes of all 7 strains examined. From that together with Fig. 3, it can be seen that although many more unique proteins were identified in strain MGΔ*pepNXOTCF_2_O_2_*, the total number of biological function groups did not increase.

### (Endo)peptidase mutants accumulate β-casein peptides that differ in physicochemical properties.

**FIG 5:**
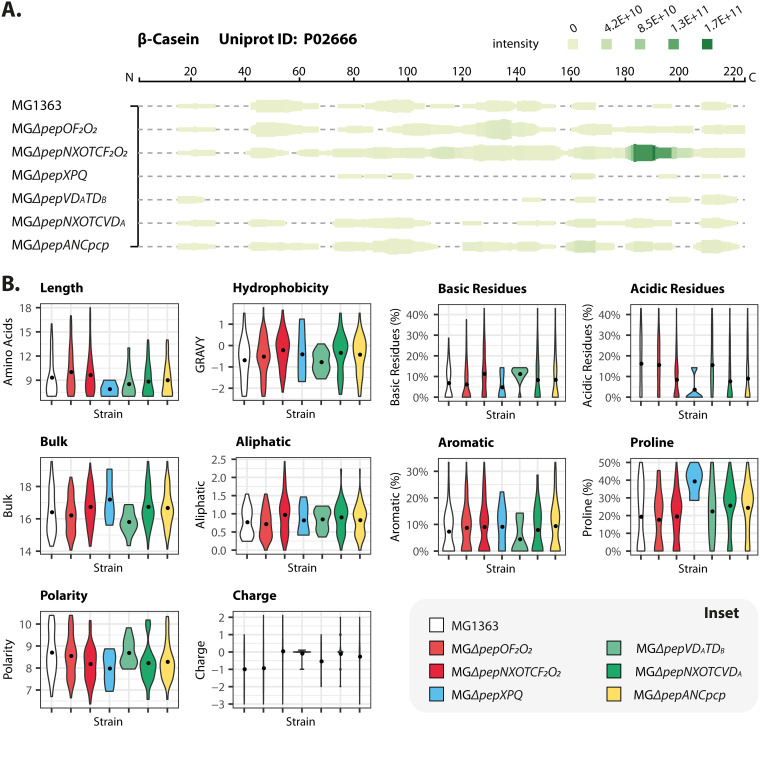
Intracellular profile and physicochemical properties of peptides derived from β-casein after its initial hydrolysis by extracellular PrtP in L. lactis MG1363 and its *pep* mutants. (A) Intracellular peptides assigned to β-casein by the PEAKS X studio software in the peptidomes of the indicated strains. The visualization tool Peptigram (30) was used to generate the figure. The linear sequence of bovine β-casein (224 amino acid residues, including the signal sequence f1-15) is shown at the top. Each vertical green bar represents an amino acid residue in β-casein, with the height denoting the count and the color intensity corresponding to the sum of peptides overlapping at this position. (B) Violin plots with mean values (black dots) showing the physicochemical properties of the intracellular peptides assigned to β-casein in the peptidomes of L. lactis MG1363 and its peptidase mutants. The inset shows the strain identity; strains are shown in the same order in each panel.

After having analyzed the intracellular peptidome profiles for the presence of peptides derived from proteins expressed by those strains, we proceeded by examining the β-casein-derived peptides therein. These peptides and their relative intensities were visualized using the web tool Peptigram. In L. lactis strain MG1363, upon digestion of β-casein by the extracellular proteinase PrtP and uptake of oligopeptides by the Opp system, peptides were retrieved that cover the majority of the β-casein sequence (Fig. 5A). Several regions in β-casein that are not represented or retrieved in the intracellular peptide pool are shown as gaps. The first 2 gaps represent the fragment from residue 1 to 15 (f1-15) and f30-40 of β-casein, which are observed in all mutants. The first gap, f1-15, is the signal peptide which exists in the β-casein precursor (UniProt P02666) which contains 224 amino acids, while the β-casein (catalog number C6905; Sigma) we used contains 209 amino acid residues (f16-224). The other 7 gaps seen in the MG1363-derived peptide pattern are covered by the peptidome of one or more of the other mutants. It is clear that all *pep* mutants produce different β-casein peptide profiles. In the intracellular peptidome of the strain lacking all endopeptidase activity, MGΔ*pepNXOTCF_2_O_2_*, the highest relative intensity (dark green area in Fig. 5A) is seen around β-casein f180-200. This strain is also the most promising mutant with respect to possessing (more) β-casein-derived putative bioactive peptides, since the identified peptides from its intracellular peptidome cover almost all parts of the β-casein molecule and at the same time have quite high intensities (Fig. 5A). It has to be noted that in mass spectrometry, peptide intensity relies on peptide ionization capacity in addition to peptide abundance, and therefore, the observed intensities cannot directly be translated to peptide concentrations. However, for the same region of β-casein, e.g., f180-200, the peptide intensity obtained with MGΔ*pepNXOTCF_2_O_2_* is dramatically higher than that obtained with the other strains. This implies that in the cytoplasm of this strain, many more peptides from this region are present than in the cytoplasm of the other strains. Peptides identified in MGΔ*pepOF_2_O_2_*, MGΔ*pepNXOTCVD_A_*, and MGΔ*pepANCpcp* also cover more of the β-casein molecule than seen in MG1363, which means that those *pep* mutants possess some β-casein-derived peptides that do not exist in the wild-type strain. Strains MGΔ*pepVD_A_TD_B_* and MGΔ*pepXPQ* produce significantly fewer PSMs and peptides (Fig. 3A), and clearly, their peptidomes also contain fewer β-casein-derived peptides.

We then examined the physicochemical properties of the peptidomes of the various strains. For the β-casein-derived peptides, those obtained with MG1363 and its *pep* mutants have very different distributions in each physicochemical property. As expected, compared to the wild type, the two endopeptidase mutants (MGΔ*pepNXOTCF_2_O_2_* and MGΔ*pepOF_2_O_2_*) contained more longer β-casein-derived peptides, while shorter peptides are present in the exopeptidase mutants (Fig. 5B, length). In agreement with its genetic makeup, the mutant in which proline-specific peptidase genes have been deleted, strain MGΔ*pepXPQ*, produces many more proline-containing peptides than all other strains (Fig. 5B, proline).

### Optimization of β-casein-derived bioactive-peptide databases.

L. lactis proteinase PrtP is a β-casein-specific caseinase. To allow identification of bioactive peptides in our peptidome data set, a comprehensive review of β-casein-derived bioactive peptides in commonly used bioactive-peptide databases was performed. The most popular of those databases for milk-derived proteins are BIOPEP, MBPDB, and EROP-Moscow. There are pros and cons for each of these databases. BIOPEP (32) has more peptide entries but does not give proper literature references for each entry. Although EROP-Moscow (33) does have literature references, these have not been updated in recent years. Also, the query page of EROP-Moscow does not support multiple sequence searches. MBPDB (34) performs best in both searching and literature updating, but it does not cover all peptides from the other two databases. For example, β-casein f(75-81) YPFPGPI is present and labeled in all 3 database as having opioid activity. BIOPEP does not provide a reference, while EROP-Moscow and MBPDB do. However, EROP-Moscow provides only the first research paper revealing the opioid activity (35), while there are 3 subsequent papers also proving the opioid activity of this peptide. In addition, 4 other bioactivities have been reported for this peptide over the past 2 decades in MBPDB: increased satiety (36), anxiety reducing (37), anticancer (38), and ACE inhibitory (6).

**FIG 6:**
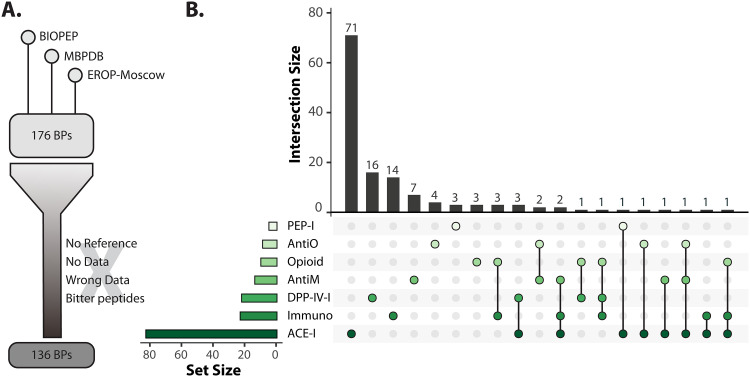
Optimization of a β-casein bioactive-peptide database. (A) Optimization strategy. BP, bioactive peptide. (B) UpSetR plot (52) highlighting the intersection of peptides with bioactivities, as indicated by the circles. Horizontal bars (set size) indicate the number of bioactive peptides for each bioactivity. The vertical bars (intersection size) show the number of peptides with a certain bioactivity activity. A peptide can have more than 1 bioactivity, as indicated by the connected circles. For example, the set size of PEP-I is 4, of which 3 peptides have only PEP-I activity while 1 peptide has both PEP-I and ACE-I activity. PEP-I, prolyl endopeptidase inhibitory; AntiO, antioxidation; AntiM, antimicrobial; DPP-IV-I, dipeptidyl peptidase IV inhibitory; Immuno, immunomodulatory; ACE-I, angiotensin-converting enzyme inhibitory.

We combined and curated these three databases by proofreading the data for each β-casein-derived bioactive peptide, excluding those for which (i) no reference was provided, (ii) a reference was given but the activity was hypothetical or predicted only (39), (iii) data were delivered but there is a discrepancy between the sequence from reference literature and the database (40), and (iv) only a bitter taste was recorded (41). Thus, we obtained 176 unique bioactive peptides, of which, after excluding those falling under definitions i to iv, 136 bioactive peptides remained (Fig. 4A). They were grouped by their bioactivities, and it is clear that more than half of the bioactive peptides have ACE-inhibitory activity (82/136 bioactive peptides). The second large activity group contains around 20 peptides with immunomodulatory or dipeptidyl peptidase IV-inhibitory (DPP-IV-I) activity. The third, much smaller, group includes peptides with antimicrobial, opioid, antioxidation, or prolyl-endopeptidase inhibitory activities. Note that 18 of the 136 peptides possess multiple bioactivities (Fig. 6B).

### L. lactis peptidase mutants produce more bioactive peptides than the wild-type strain.

Table 2 summarizes the bioactive peptides identified through nanoLC-MS/MS in the intracellular peptidomes of each strain by comparing the identified β-casein-derived peptides to our curated bioactive-peptide database. As expected on the basis of the results presented above, MGΔ*pepNXOTCF_2_O_2_* is the most promising strain, as 18 bioactive peptides could be identified in its intracellular peptidome. Most of the peptides have ACE-inhibitory activity (11/18), while other bioactivities are also found, such as DPP-IV-inhibitory, antioxidative, antimicrobial, and immunoregulatory activities. Strains MGΔ*pepNXOTCVD_A_* and MGΔ*pepANCpcp* are second with respect to the number of bioactive peptides that are obtained with these strains. Most of the β-casein-derived peptides identified in both strains have the same sequence (7/9). All β-casein-derived bioactive peptides identified in the peptidome of MGΔ*pepNXOTCVD_A_* are also present in that of MGΔ*pepNXOTCF_2_O_2_*. As for MGΔ*pepANCpcp*, except for AVPYPQR, the other 8 bioactive peptides are also observed in MGΔ*pepNXOTCF_2_O_2_*. The β-casein-derived peptidome of MGΔ*pepOF_2_O_2_* contains 6 bioactive peptides; except for peptide VPVEPFTE, the other 5 peptides are also present in the samples of MGΔ*pepOF_2_O_2_*. No bioactive peptides were observed when using MGΔ*pepVD_A_TD_B_* to degrade β-casein, and only 2 were found when employing strain MGΔ*pepXPQ*. As explained above, these might be caused by the disruption of other biological processes, such as peptidoglycan biosynthesis, when deleting the dipeptidase PepV (31).

**TABLE 2:** Bovine β-casein-derived bioactive peptides identified in the intracellular peptidomes of L. lactis MG1363 and its peptidase knockout mutants

Strain and peptide sequence	β-Casein fragment (start–end)	Theoretical mass (Da)	Mass error (ppm)^*a*^	Reported bioactivity^*b*^	Reference(s)
MG1363					
YPFPGPIPN	75–83	1,000.5018	0.9/3.4/−1.8	ACE-I, DPP-IV-I, opioid	56–58
LPQNIPP	85–91	777.4385	3.3/4.9/3.6	DPP-IV-I	57
LPQNIPPL	85–92	890.5225	3.1/—/−0.5	DPP-IV-I	57
TQTPVVVPPFLQPE	9–106	1,550.8344	3.6/6.8/—	Anti-O	59
MGΔ*pepOF_2_O_2_*					
YPFPGPIPN	75–83	1,000.5018	3.3/2.8/0	ACE-I, DPP-IV-I, opioid	56–58
HKEMPFPK	121–128	1,012.5164	0.3/−2/−1.3	Anti-M	60
YPVEPFTE	129–136	980.4491	9.7/8/4.3	ACE-I	61
SQSKVLPVPQ	181–190	1,081.6132	6.7/6.9/5.4	ACE-I	9
RDMPIQAF	198–205	976.4800	—/6.1/5	ACE-I	40
QEPVLGPVRGPFPIIV	209–224	1,716.9926	4.8/6.1/2.5	ACE-I	62
MGΔ*pepNXOTCF_2_O_2_*					
LNVPGEIVE	21–29	968.5178	2.5/3.8/2.5	ACE-I	10
VYPFPGPIPN	74–83	1,099.5702	1.3/2/3.2	ACE-I, anti-O	63
LVYPFPGPIPNSLPQ	73–87	1,637.8817	3.6/3.8/—	ACE-I, PEP-I	64
LPQNIPPL	85–92	890.5225	2.8/1.5/4	DPP-IV-I	57
PQNIPPL	86–92	777.4385	2.9/2/2.2	DPP-IV-I	57
NIPPLTQTPV	88–97	1,078.6023	4.2/4.6/4/6	ACE-I	10
TQTPVVVPPFLQPE	93–106	1,550.8344	2.5/2.5/2.5	Anti-O	59
VKEAMAPK	113–120	872.4789	−2.9/−4.1/−1.6	Anti-O, anti-M	65
HKEMPFPK	121–128	1,012.5164	5.2/2.9/4.1	Anti-M	60
LHLPLPL	148–154	801.5112	2.8/5.5/6.1	ACE-I	11
NLHLPLPLL	147–155	1,028.6382	1.9/3.8/3.9	ACE-I	66
SQSKVLPVPQ	181–190	1,081.6132	3.8/5.1/4.5	ACE-I	9
KVLPVPQK	184–191	907.5854	5.1/4.5/7	Anti-O	65
KVLPVPQ	184–190	779.4905	3.6/1.8/9.1	ACE-I	67
VLPVPQK	185–191	779.4905	4.7/6.9/7.6	Anti-M, anti-O	65
RDMPIQAF	198–205	976.4800	4.7/3.8/4.9	ACE-I	40
YQEPVLGPVRGPFPIIV	208–224	1,880.0559	4.3/4.3/—	ACE-I, anti-M, immuno-R	68–70
QEPVLGPVRGPFPIIV	209–224	1,716.9926	2.6/2.7/2.2	ACE-I	62
MGΔ*pepXPQ*					
YPFPGPIPN	85–83	1,000.5018	−0.6/−1.4/−0.3	ACE-I, DPP-IV-I, opioid	56–58
LPQNIPPL	85–92	890.5225	1.5/2.8/—	DPP-IV-I	57
MGΔ*pepVD_A_TD_B_*					
None					
MGΔ*pepNXOTCVD_A_*					
LNVPGEIVE	21–29	968.5178	—/1.9/2	ACE-I	10
VYPFPGPIPN	74–83	1,099.5702	—/2.5/3.4	ACE-I, anti-O	63
LPQNIPPL	85–92	890.5225	—/3.9/4.9	DPP-IV-I	57
PQNIPPL	86–92	777.4385	—/1.7/4.1	DPP-IV-I	57
NIPPLTQTPV	88–97	1,078.6023	—/3.9/5.5	ACE-I	10
TQTPVVVPPFLQPE	93–106	1,550.8344	—/3.6/4.7	Anti-O	59
LHLPLPL	148–154	801.5112	—/5.8/3.5	ACE-I	11
SQSKVLPVPQ	181–190	1,081.6132	—/4.5/4.3	ACE-I	9
KVLPVPQ	184–190	779.4905	1.4/0.8/2.9	ACE-I	67
MGΔ*pepANCpcp*					
LNVPGEIVE	21–29	968.5178	2.9/6.4/4.2	ACE-I	10
VYPFPGPIPN	74–83	1,099.5702	5.4/5.5/4.8	ACE-I, anti-O	63
NIPPLTQTPV	88–97	1,078.6023	4.7/8/10	ACE-I	10
TQTPVVVPPFLQPE	93–106	1,550.8344	6.2/8.2/6.7	Anti-O	59
HKEMPFPK	121–128	1,012.5164	0.1/1.2/−0.1	Anti-M	60
LHLPLPL	148–154	801.5112	5.2/9.4/6.7	ACE-I	11
SQSKVLPVPQ	181–190	1,081.6132	4.7/4.9/6.5	ACE-I	9
KVLPVPQ	184–190	779.4905	4.1/1.8/3.9	ACE-I	67
AVPYPQR	192–198	829.4446	2.9/1.4/—	ACE-I, anti-M, anti-O	60, 65, 71

a The mass error is calculated as 10^6^ × (observed mass − theoretical mass)/theoretical mass. The three numbers in the mass error column represent the biological triplicates; a dash (—) signifies that the peptide was not detected in one of the triplicates.

b ACE-I, angiotensin-converting enzyme inhibitory; anti-M, antimicrobial; anti-O, antioxidative; DPP-IV-I, dipeptidyl peptidase 4 inhibitory; immuno-R, immunoregulatory.

## DISCUSSION

In this study, we present an analytical framework consisting of peptidome extraction followed by tandem mass spectrometric identification and bioinformatic analysis to untangle the intracellular peptidome of L. lactis and to assess the potential of this organism as a cell factory for the production of bioactive peptides. The developed protocol is reproducible and can be performed in less than 2 h from peptidome extraction to mass spectrometric analysis.

The quality of peptide identification relies on a suitable searching algorithm. PEAKS studio identified more peptides in our data sets than all the other search engines from SearchGUI (Fig. 2C), while it is also user-friendly because of its well-designed interface. However, when this commercial tool is not accessible because of its price, SearchGUI could be a good alternative because it includes the mainstream open-source search engines. MS-GF+ and Andromeda are the top algorithms in the proteomics/peptidomics field. MS-GF+ delivered relatively good and reproducible identification results. However, unlike PEAKS, it does not provide the relative intensities of identified peptides, precluding a visualization of β-casein digestion profiles as presented in Fig. 5A. Thus, for data analysis consistency, we did not combine the results from PEAKS and MS-GF+. Andromeda, the search engine of MaxQuant, is designed for large mass spectrometric data sets but is geared mostly toward human proteomes/peptidomes and is unsuitable for our bacterial peptidomics data. Here, we focused on identifying bioactive peptides, which normally contain 2 to 20 amino acid residues (42). Note that the oligopeptide transport system (Opp) of L. lactis possesses the capacity to transport peptides from 4 up to at least 18 residues (43). We therefore set the mass spectrometry detection window to 170 to 2,000 Da, which generally covers peptides containing 2 to 18 amino acid residues, considering that the average molecular weight of an amino acid is 110 Da. The gap regions in the β-casein peptide profile of each strain (Fig. 5A) might be due to the fact that some peptides have more than 18 amino acids residues and are thus beyond our detection window.

**FIG 7:**
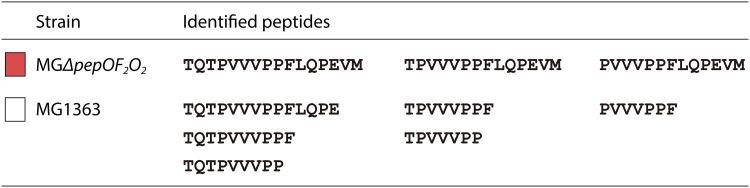
β-Casein-derived peptides identified in the intracellular peptidomes of MGΔ*pepOF_2_O_2_* (peptide sequences in red) and MG1363 (peptide sequences in black).

The fact that the β-casein-derived bioactive peptides identified in the intracellular peptidome of each strain treated under the same conditions differs demonstrates the potential of these *pep* mutants. The β-casein-derived peptides obtained with the wild-type strain MG1363 cover the whole protein (Fig. 5A), with bioactive peptides originating only from β-casein f(70-110) (Table 2). Novel peptides are found when employing the *pep* mutant strains, which shows the potential of β-casein as a bioactive-peptide source in combination with L. lactis serving as an enzyme complex. The L. lactis peptidases have been classified in different groups on the basis of their cleavage specificity. For example, enzymes in the endopeptidase group will cleave internally in an oligopeptide, and knocking them all out should result in the accumulation of relatively longer peptides. Take peptide TQTPVVVPPFLQPEVM in Fig. 7 as an example. It might be that in MG1363, peptide TQTPVVVPPFLQPEVM was internalized and hydrolyzed into TQTPVVVPPFLQPE/VM, TQTPVVVPPF/LQPEVM, and TQTPVVVPP/FLQPEVM. The longer N-terminal parts in each case were detected, while the C-terminal parts were not. This may be due to the detection limit of the LC/MS equipment (VM) or because the C-terminal peptides were degraded further by other intracellular peptidases (LQPEVM or FLQPEVM). In mutant MGΔ*pepOF_2_O_2_*, lacking the three endopeptidases, TQTPVVVPPFLQPEVM would not be degraded, leading to its accumulation (Fig. 7). In fact, the three peptides TQTPVVVPPFLQPEVM, TPVVVPPFLQPEVM, and PVVVPPFLQPEVM were detected only in the mutants MGΔ*pepOF_2_O_2_* and MGΔ*pepNXOTCF_2_O_2_*, which both lack all endopeptidases. In some other mutants, smaller derivative peptides identical to those in MG1363 can be observed; i.e., TQTPVVVPP and PVVVPPF were also identified in strains MGΔ*pepNXOTCVD_A_* and MGΔ*pepANCpcp*, respectively, while TPVVVPP was also identified in strain MGΔ*pepXPQ*. These observations indicate that one or more of the endopeptidases prefers cutting C-terminal glutamic acid, phenylalanine, and proline residues, which coincides with a previous study showing that PepF can (likes to) cut C-terminal proline and phenylalanine (44).

On the one hand, this presence/absence of certain (groups of) peptidases might liberate interesting bioactivities from the β-casein molecule, and on the other hand, it might release enough free amino acids so that L. lactis growth and functioning are not severely affected. When a group of specialty peptidases is removed, a problem might arise if they not only are responsible for β-casein digestion but also are important in another metabolic pathway(s), disrupting certain essential processes. A clear example is the role that PepV plays in peptidoglycan synthesis (31).

Fermentation is an easy and cost-effective method to generate bioactive peptides in fermented milk products. This study presents a comprehensive analysis of the L. lactis intracellular peptidome after *in vivo* β-casein degradation. The work suggests that the number of different bioactive peptides and the bioactivity diversity can be increased by editing the proteolytic system of this LAB starter strain. L. lactis MGΔpep*NXOTCF_2_O_2_* has the best performance in producing peptides with high intensities among peptides that have a variety of bioactivities. It might thus potentially be useful as a bioactive-peptide cell factory. The fact that the peptides are intracellular should make them less sensitive to, for instance, digestive enzymes. Our work could also be used as a guideline for modifying proteolytic systems in other LAB and for further analyzing and visualizing the intracellular proteome/peptidome data to explore their potential as peptide cell factories.

## MATERIALS AND METHODS

### Bacterial strains and culture conditions.

The bacterial strains used in this study are listed in Table 1. Lactococcus lactis MG1363 and its derivatives were cultivated in M17 medium (catalog number DF1856-17-4; BD Difco, Detroit, MI, USA) containing 0.5% (wt/vol) glucose (GM17) at 30°C. Erythromycin (catalog number E6376; Sigma-Aldrich, Santa Clara, CA, USA) was added at a final concentration of 5 μg/ml when required. Chemically defined SA medium with 0.5% (wt/vol) glucose and 20 μg/ml 5-fluoroorotic acid (5-FOA) (catalog number F5013; Sigma-Aldrich, Santa Clara, CA, USA) as a sole pyrimidine source was used for the generation of chromosomal knockouts, as described previously (17). Escherichia coli DH5α was used for cloning purposes; it was cultivated aerobically at 37°C in LB medium (catalog number LMM01; Formedium, Norfolk, UK) with erythromycin at a final concentration of 200 μg/ml when required.

### Recombinant DNA techniques and oligonucleotides.

Standard molecular cloning techniques were performed essentially as described previously (45). Chromosomal DNA from L. lactis was isolated using the GenElute bacterial genomic DNA kit (catalog number NA2110-1KT; Sigma-Aldrich, Santa Clara, CA, USA). Plasmids and PCR products were isolated and purified using the NucleoSpin Plasmid EasyPure kit (catalog number MN 740727.250; Macherey-Nagel, Leiden, The Netherlands) and the NucleoSpin Gel & PCR Clean-up kit (catalog number MN 740609.250; Macherey-Nagel, Leiden, The Netherlands), respectively, according to the manufacturer’s instructions. PCRs for cloning purpose were performed with Phusion high-fidelity DNA polymerase (catalog number F530L; Thermo Fisher Scientific, MA, USA) according to the manufacturer’s protocol. Enzymes were purchased from Fermentas (Thermo Fisher Scientific, MA, USA) and New England Biolabs (Ipswich, MA, USA). Colony PCRs were performed with homemade *Pfu* polymerase. Inserts and linearized vector were fused using the Quick-Fusion cloning kit (catalog number B22612; BioConnect) according to the manufacturer with the modification that half of the recommended volume per reaction was used. Oligonucleotides employed in this study are listed in Table S1 in the supplemental material and were purchased from Biolegio BV (Nijmegen, The Netherlands). Competent E. coli cells were transformed using heat shock (46), while electrocompetent L. lactis cells were transformed using electroporation (47) with a Bio-Rad Gene Pulser (Bio-Rad Laboratories, CA, USA). All nucleotide sequencing was performed at Macrogen Europe (Amsterdam, The Netherlands).

### Construction of integration plasmids for knocking out peptidase genes from L. lactis MG1363.

All plasmids that were used or constructed during this study are listed in Table 3. Relevant regions of all plasmids were sequenced to confirm their nucleotide sequences. All integration plasmids were constructed using the same workflow that is described here for only one, pCH001, as an example, as follows. Linearized vector pCS1966 was amplified using primers pCS1966_1FW/pCS1966_1RV. Primer pairs pCH-0017/pCH-0018 and pCH-0019/pCH-0020 were used, respectively, to obtain upstream (UP_F2) and downstream (DOWN_F2) regions of peptidase gene *pepF_2_*. Primer pair pCH-0017/pCH0020 was used to perform an overlap PCR to obtain the flanking region UP+DOWN_F2. Primers pCH-0017 and pCH-0020 contain 15 nucleotides at one end, overlapping with the sequence on the 5′ end of the linearized vector, followed by the flanking region of *pepF_2_* gene and 15 nucleotides overlapping with the sequence on the 3′ end of the linearized vector. The fragment UP+DOWN_F2 was fused with the linearized vector using Quick-Fusion, after which the reaction mixture was directly used to transform competent E. coli DH5α. The resulting vector was designated pCH001. Primers pCH-0083/0099/0100 were used for colony PCR and nucleotide sequencing confirmation.

**TABLE 3:** Plasmids used in this study

Plasmid	Host(s)	Description	Antibiotic resistance	Reference
pCS1966	E. coli	L. lactis integration vector	Ery^*a*^	17
pTLR	E. coli	L. lactis expression shuttle vector	Ery	Lab collection
pLP712	L. lactis	Prt^+^ Lac^+^, 54-kb proteinase/lactose plasmid of NCDO712		53
pCS1966	E. coli	L. lactis integration vector	Ery	This study
pCH001	E. coli	L. lactis integration vector, knockout *pepF_2_*	Ery	This study
pCH002	E. coli	L. lactis integration vector, knockout *pepO_2_*	Ery	This study
pCH003	E. coli	L. lactis integration vector, knockout *pepA*	Ery	This study
pCH004	E. coli	L. lactis integration vector, knockout *pepP*	Ery	This study
pCH005	E. coli	L. lactis integration vector, knockout *pepV*	Ery	This study
pCH006	E. coli	L. lactis integration vector, knockout *pepM*	Ery	This study
pCH007	E. coli	L. lactis integration vector, knockout *pcp*	Ery	This study
pCH008	E. coli	L. lactis integration vector, knockout *pepQ*	Ery	This study
pCH009	E. coli	L. lactis integration vector, knockout *pepD_A_*	Ery	This study
pCH010	E. coli	L. lactis integration vector, knockout *pepF_1_* in pLP712	Ery	This study
pCH011	E. coli	L. lactis integration vector, knockout *pepO*	Ery	This study
pCH012	E. coli	L. lactis integration vector, knockout *pepC*	Ery	This study
pCH013	E. coli	L. lactis integration vector, knockout *pepN*	Ery	This study
pCH014	E. coli	L. lactis integration vector, knockout *pepX*	Ery	This study
pCH015	E. coli	L. lactis integration vector, knockout *pepT*	Ery	This study
pCH016	E. coli	L. lactis integration vector, knockout *pepD_B_*	Ery	This study
pCH020	E. coli, L. lactis	pTLR-PrtPM, for expression of protease PrtP and PrtM under its own promoter	Ery	This study

a Ery, erythromycin.

### Construction of L. lactis (multi)peptidase knockout mutants.

All peptidase gene knockout strains were made using the same workflow that is described here only for the construction of the multiple endopeptidase mutant strain CH018 as an example, as follows. Integration plasmid pCH011, a *pepO* knockout plasmid, was introduced in MG1363 via electroporation. Knockout mutants were obtained by a two-step homologous recombination strategy (17), First, plasmid chromosomal integrates were selected on erythromycin-containing GM17 plates. Subsequently, the marker-free knockout strain was obtained through counterselection on 5-FOA on SA medium plates. The resulting strain, CH011 (MGΔ*pepO*), underwent the same 2-step recombination protocol using pCH001 to obtain the peptidase double mutant strain CH017 (MGΔ*pepOF_2_*). Strain CH018 (MGΔ*pepOF2O2*) was obtained using the strategy with plasmid pCH002 on strain CH017. All relevant chromosomal regions of each deletion strain were confirmed by nucleotide sequencing.

### Construction of plasmid pCH020 for expressing proteinase PrtP in L. lactis MG1363.

The flanking regions of the *prtPM* genes from plasmid pLP712 (15) were amplified together using primers pCH-0173/pCH-0174. The fragment was ligated into plasmid pTLR employing NcoI/XhoI restriction sites. The resulting plasmid was named pCH020.

### β-Casein degradation *in vivo*.

*In vivo* β-casein breakdown was examined using the method of Kunji et al. (48) with the following modifications. An overnight culture was diluted to a starting optical density at 600 nm (OD_600_) of 0.05 in 50 ml of GM17 with 5 μg/ml erythromycin, when required. The culture was grown at 30°C and when the OD_600_ reached 0.7, which corresponds to the early exponential growth phase, the cells were collected by centrifugation at 6,000 × *g* for 5 min. They were washed twice with wash buffer (100 mM morpholineethanesulfonic acid [MES]-KOH [pH 6.5] with 2 mM CaCl_2_) to prevent autoproteolysis and release of the proteinase PrtP. Cells were then concentrated to an OD_600_ of 14 and resuspended in 2 ml of 4-mg/ml β-casein (catalog number C6905; Sigma-Aldrich, Santa Clara, CA, USA) in wash buffer with 0.5% (wt/vol) glucose. The suspension was incubated for 3 h at 30°C with slow rotation (10 rpm) in a rotator incubator oven (catalog number G2545A; Agilent Technologies, Inc., CA, USA). Cells were then spun down at 12,000 × *g* for 3 min, after which both the supernatant and the cells were saved at −80°C until further use.

### Cytoplasmic peptidome extract preparation.

Frozen cells incubated with β-casein were thawed and resuspended in 2 ml 1 M LiCl in 50 mM Tris (pH 8.0) to extract proteins in the surface layer or anchored to the cell wall through noncovalent interactions (49). After incubation at 4°C for 1 h, the cells were spun down at 4,000 × *g* for 10 min. The supernatant, named LiCl extract, was saved at −80°C. The cells were washed twice with MilliQ water (Millipore, MA, USA) and subsequently disrupted in a mini-beadbeater (catalog number 112011EUR; BioSpec, OK, USA) using 3 cycles of 1 min on and 1 min off. Disrupted cells were spun down at 11,000 × *g* at 4°C for 10 min. The supernatant was collected and filtered through a 0.2-μm-pore-size filter (catalog number 41055511; Boom BV, Meppel, The Netherlands) and then ultrafiltrated through Amicon 3-kDa-molecular-weight-cutoff membranes (catalog number UFC500324; Millipore, MA, USA). The pool of peptides less than 3 kDa was collected and saved at −80°C until further use.

### nanoLC-MS/MS.

All samples were analyzed at the Interfaculty Mass Spectrometry Center, University of Groningen, on a nanoLC-MS/MS consisting of an Ultimate 3000 LC system (Dionex, Amsterdam, The Netherlands) interfaced with a Q-Exactive Plus mass spectrometer (Thermo Fisher Scientific, MA, USA). Peptide mixtures were loaded onto a 5-mm by 300-μm (inner diameter) C_18_ PepMAP100 trapping column (Thermo Fisher Scientific, MA, USA) with 2% acetonitrile in 0.1% formic acid at 20 μl/min. After loading and washing for 3 min, peptides were eluted onto a 15-cm by 75-μm (inner diameter) C_18_ PepMAP100 nanocolumn (Dionex, Amsterdam, The Netherlands). A mobile-phase gradient at a flow rate of 300 nl/min and with a total run time of 75 min was used: 2% to 50% of solvent B in 60 min, 50% to 90% B in 1 min, 90% B during 13 min, and back to 2% B in 1 min (held for 15 min). Solvent A was 100:0 water-acetonitrile (vol/vol) with 0.1% formic acid, and solvent B was 0:100 water-acetonitrile (vol/vol) with 0.1% formic acid. In the nanospray source a stainless-steel emitter (Thermo Fisher Scientific, MA, USA) was used at a spray voltage of 1.8 kV with no sheath or auxiliary gas flow. The ion transfer tube temperature was 275°C. Spectra were acquired in data-dependent mode with a survey scan at *m/z* 300 to 1650 at a resolution of 70,000, followed by MS/MS fragmentation of the top 10 precursor ions. Singly charged ions were excluded from MS/MS experiments, and fragmented precursor ions were dynamically excluded for 20 s. PEAKS studio version X software (Bioinformatics Solutions, Inc., Waterloo, Canada) was used to search the MS data against a protein sequence database of the L. lactis MG1363 proteome (UniProt database) to which the sequence of β-casein (UniProt P02666) was added. Search parameters were as follows: no enzyme specificity, fixed modification, carbamidomethylation of cysteine, variable modifications, oxidation of methionine and phosphorylation of serine, precursor mass tolerance of 15 ppm, and fragment mass tolerance of 0.02 Da. The false-discovery rate was set at 0.1%.

### Data analysis and visualization.

The peptide spectrum matches (PSMs), identified peptides, and identified protein number were obtained from PEAKS. The identified peptides were exported for further analysis in R by using the Pheatmap R package. Venn diagrams were made using the webtool Calculate and Draw Custom Venn Diagrams (http://bioinformatics.psb.ugent.be/webtools/Venn/). Gene ontology analysis was performed using Gene Set Enrichment Analysis (GSEA) provided by the GENOME2D software available at http://gseapro.molgenrug.nl/. Circos plots were made using the Circlize R package (50). For further peptidomic analysis, peptide sequences of biological triplicates were combined, and only unique peptides present at least twice among triplicates were used. Thus, 7 data sets of all 21 samples were generated. The further data analysis mentioned below was performed on these combined data sets. Profiles of β-casein-derived peptides in the L. lactis intracellular peptidome were visualized by the web-based tool Peptigram (30). Data of the parameter relative intensity were generated from the average intensity of the replicates of each chosen peptide. Peptide physicochemical properties were computed using the aminoAcidProperties function of the R package “alakazam,” version 0.2.8 (51). The proportion of proline in each peptide was manually calculated. The UpSet plot of our optimized bioactive-peptide database was generated using the UpSetR package (52).

## Supplementary Material

Supplemental file 1
