# Supplementary material for: Editing of the Proteolytic System of Lactococcus lactis Increases Its Bioactive Potential
Source: Appl Environ Microbiol. 2020 Sep 1;86(18):e01319-20. doi: 10.1128/AEM.01319-20 (PMC7480361; doi:10.1128/AEM.01319-20)
Supplement: Supplemental file 1 [file AEM.01319-20-s0001.pdf]

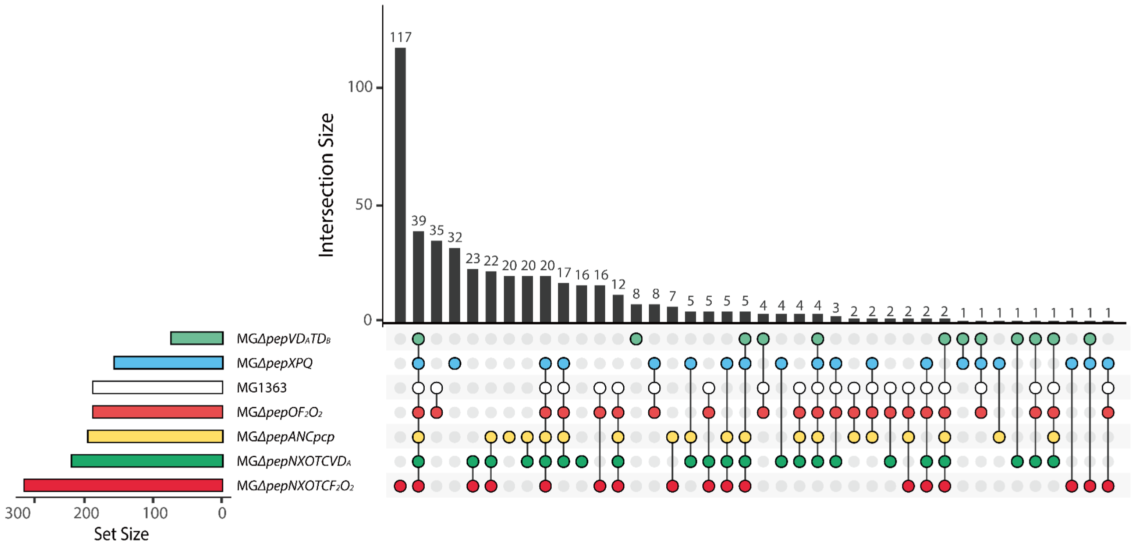

Suppl Fig 1 UpSetR plot (1) **highlighting the intersection of identified proteins from each strain, as indicated by the bullet points.** Horizontal bars (Set size) indicate the number of identified proteins for each strain. The vertical bars (Intersection size) show the number of proteins from certain strain(s). A protein can be detected in more than 1 strain, as indicated by the connected bullet points.

1. Conway JR, Lex A, Gehlenborg N. 2017. UpSetR: an R package for the visualization of intersecting sets and their properties. Bioinformatics 33:2938–2940.

12 **Suppl. Table 1. Oligonucleotides used in this study.**

Short Name	Long Name	Sequence (5′ → 3′′)
pCS1966_1FW	pCS1966_1FW	GTGCCTAATGAGTGAGCTAACTC
pCS1966_1RV	pCS1966_1RV	GTGGAATTGTGAGCGGATAAC
pCH-0017	17-F2_Up_FW	CGCTCACAATTCCACCTAAAGAGCTGCAATTTATGGT
pCH-0018	18-F2_Up_RV	AAAGCCAGTTGCATATTGAACTTCTCTAATTCTGTTTCC
pCH-0019	19-F2_Down_FW	GGAAACAGAATTAGAGAAGTTCAATATGCAACTGGCTTT
pCH-0020	20-F2_Down_RV	TCACTCATTAGGCACTTCCAAAGCTCGTTGATTTCT
pCH-0021	21-O2_UP_FW	CGCTCACAATTCCACGTCACGACCGAGTATCTTCTTG
pCH-0022	22-O2_UP_RV	GGACCAGCTTCAGTTTCAATGTTGTCAGCTGGAATTTGTG C
pCH-0023	23-O2_DOWN_FW	GCACAAATTCCAGCTGACAACATTGAAACTGAAGCTGGTC C
pCH-0024	24-O2_DOWN_RV	TCACTCATTAGGCACGGCTGAAATTACCGAAAATACAA
pCH-0025	25-A_UP_FW	CGCTCACAATTCCACCCGACGTTTGTGTTATATGTT
pCH-0026	26-A_UP_RV	ATGGCTATGGATATAGCGTTCTGGTTGATAGCCTAGC
pCH-0027	27-A_DOWN_FW	GCTAGGCTATCAACCAGAACGCTATATCCATAGCCAT
pCH-0028	28-A_DOWN_RV	TCACTCATTAGGCACGAACCTTGGTATATCGTTGAT
pCH-0029	29-P_UP_FW	CGCTCACAATTCCACCTGACCGACCATAATATTTTGAC
pCH-0030	30-P_UP_RV	CATCTTCAACTGTACCCGCTGTTCCAGAA
pCH-0031	31-P_DOWN_FW	GTACAGTTGAAGATGACTTATTAGTGACAG
pCH-0032	32-P_DOWN_RV	TCACTCATTAGGCACCTGACGAAGTCTTAAAACTTCA
pCH-0033	33-V_UP_FW	CGCTCACAATTCCACCGAGAAATAGACTTAGCGTT
pCH-0034	34-V_UP_RV	TCGCTTGGTTGTATAACCATCACGTTCG
pCH-0035	35-V_DOWN_FW	TATACAACCAAGCGAATGAAATGAAACCT
pCH-0036	36-V_DOWN_RV	TCACTCATTAGGCACTTAGTCACCAGATGATTTCGT
pCH-0037	37-M_UP_FW	CGCTCACAATTCCACCAAGAGGTCTCTTATGCTTGA
pCH-0038	38-M_UP_RV	ATGGTTAAGACCATTCCTATCACGAAGACCAATATG
pCH-0039	39-M_DOWN_FW	CATATTGGTCTTCGTGATAGGAATGGTCTTAACCAT

Short Name	Long Name	Sequence (5′ → 3′′)
pCH-0040	40-M_DOWN_RV	TCACTCATTAGGCACTGTTCCAGGCATCGTATATC
pCH-0041	41-pcp_UP_FW	CGCTCACAATTCCACCTTAGAAGCCAATTACTCTGTT
pCH-0042	42-pcp_UP_RV	GTTTCGATATCGCCTTTGGCTTCAATCGCAGGATTA
pCH-0043	43-pcp_DOWN_FW	TAATCCTGCGATTGAAGCCAAAGGCGATATCGAAAC
pCH-0044	44-pcp_DOWN_RV	TCACTCATTAGGCACATTTCACCATTACAATCAAAGG
pCH-0045	45-Q_UP_FW	CGCTCACAATTCCACCATTTCCATTCACAACGCGAC
pCH-0046	46-Q_UP_RV	GGCAATCCTCAATACGAACGCACCAAATTGTGCTTTCAAA
pCH-0047	47-Q_DOWN_FW	GGTTTGAAAGCACAATTTGGTGCGTTCGTATTGAGGATTGCC
pCH-0048	48-Q_DOWN_RV	TCACTCATTAGGCACCAAGTGGCCAAAGAAAGCTC
pCH-0049	49-DA_UP_FW	CGCTCACAATTCCACCCACCGAGTCTACTTGATATT
pCH-0050	50-DA_UP_RV	AACTGCTTCAAGGGATTGTGCAATTAGAGTGCTACC
pCH-0051	51-DA_DOWN_FW	GGTAGCACTCTAATTGCACAATCCCTTGAAGCAGTT
pCH-0052	52-DA_DOWN_RV	TCACTCATTAGGCACAAGATCAGTTAGAAAAAATGCTG
pCH-0053	53-F1_UP_FW	CGCTCACAATTCCACACGGGTTAATGAACAATAAATG
pCH-0054	54-F1_UP_RV	TCAACTCGCTGAATCAAGTAAATGACCA
pCH-0055	55-F1_DOWN_FW	GATTCAGCGAGTTGATATGGAATCTACCGA
pCH-0056	56-F1_DOWN_RV	TCACTCATTAGGCACAAGTACTGACACGTGCATAC
pCH-0057	57-O_UP_FW	CGCTCACAATTCCACACTTAGGGCACTATGATGCTA
pCH-0058	58-O_UP_RV	TTGGCACTCTGCAGGAATTTCTGCATT
pCH-0059	59-O_DOWN_FW	CCTGCAGAGTGCCAATATTCCTCCAACA
pCH-0060	60-O_DOWN_RV	TCACTCATTAGGCACTGAAGTGATCCAACATGCTT
pCH-0061	61-C_UP_FW	CGCTCACAATTCCACTGGATAATCGGGGACTACAAT
pCH-0062	62-C_UP_RV	GCAACGACACTTCGAGTGATGAAAGCA
pCH-0063	63-C_DOWN_FW	TCGAAGTGTCGTTGCTTCTGATGAATGG
pCH-0064	64-C_DOWN_RV	TCACTCATTAGGCACCTCGTCAGCTGTAAAATCTCT
pCH-0065	65-N_UP_FW	CGCTCACAATTCCACAAGGAGGAGCCTATCATGAA

Short Name	Long Name	Sequence (5′ → 3′′)
pCH-0066	66-N_UP_RV	CAGCAGTTGAAGTGTAATCCTTTGGTATG
pCH-0067	67-N_DOWN_FW	ACACTTCAACTGCTGATAAATTAGCGGA
pCH-0068	68-N_DOWN_RV	TCACTCATTAGGCACTGTCATGGATGAAAGCCTTC
pCH-0069	69-X_UP_FW	CGCTCACAATTCCACGAACCTTTAGCTTCACCCAA
pCH-0070	70-X_UP_RV	GTAAACTAAGCTGATCAAGTTCTGCTA
pCH-0071	71-X_DOWN_FW	ATCAGCTTAGTTTACTGACAGTCAGTGAT
pCH-0072	72-X_DOWN_RV	TCACTCATTAGGCACATTCTGGATGCCATTGAACA
pCH-0073	73-T_UP_FW	CGCTCACAATTCCACAAGCAAGTTGTTCCACAATT
pCH-0074	74-T_UP_RV	GTTTGTACAGAAACAAATTCAACAAGAGCTTTCATATCTT CA
pCH-0075	75-T_DOWN_FW	TGAAGATATGAAAGCTCTTGTTGAATTTGTTTCTGTACAA AC
pCH-0076	76-T_DOWN_RV	TCACTCATTAGGCACTAACATTGAGCGAAAGAACG
pCH0328	0328-DB-Up_F	CGCTCACAATTCCACGTGAATGGCGCAATGGTCTGACTT
pCH0329	0329-DB-Up_R	AAGAACGACCATACGTCCATCATCATTACGGGCAATCATTG
pCH0330	0330-DB-Down_F	TGCCCGTAATGATGATGGACGTATGGTCGTTCTTGGTTCAG
pCH0331	0331-DB-Down_R	TCACTCATTAGGCACCACTCCAGCCTTCTTCATTGAGTCT A
pCH-0083	83-Seq_mid_01_F2	GTCGATTCATCATGGGGTTAC
pCH-0084	84-Seq_mid_02_O2	GACTTCCTTGGTGGCGTGTA
pCH-0085	85-Seq_mid_03_A	GTCACAGTTAAACTAGCAAATG
pCH-0087	87-Seq_mid_05_V	GTACTTTTCTAGCTCCATTGTTG
pCH-0089	89-Seq_mid_07_pcp	CGGAAATTATCCCCATCATAGC
pCH-0091	91-Seq_mid_09_DA	GATGAATCCAGCACGTGAC
pCH-0094	94-Seq_mid_12_C	GCATCTCTCATATGTTTGACGA
pCH-0095	95-Seq_mid_13_N	GCACTTACACGCGTTATCG
pCH-0097	97-Seq_mid_15_T	GAGAGACCTACGAGTGAGAG
pCH-0099	0099-Seq_F_pCS1966	CTGCAGGAATTCGATATCAAGC
pCH-0100	0100-Seq_R_pCS1966	CTTTGAGTGAGCTGATACCGC

Short Name	Long Name	Sequence (5′ → 3′′)
pCH-0101	0101-PrtP_F	GTCTGTAAACGGCTAAATAATAACG
pCH-0102	0102-PrtP_R	AACAATGTGAAGTCAGCTGCCTAA
pCH-0103	0103-PrtP_1	GCAAAACTTGGTATGCCGACAT
pCH-0104	0104-PrtP_2	AAGTACCACTGAACCAGCCAAA
pCH-0105	0105-PrtP_3	TATGGTGACAATACCATCAAGGT
pCH-0106	0106-PrtP_4	CTTGATGCCACCAAGAGCGTTA
pCH-0107	0107-PrtP_5	CGCAGATTGAATTCACACTATCT
pCH-0108	0108-PrtP_6	CAGCACACCCGGATGATAGTC
pCH-0109	0109-PrtP_7	CTCTGACACTTCTGCAACAACC
pCH-0110	0110-PrtP_8	TCAGGCAAGCGTTAAAGCAGCT
pCH-0111	0111-PrtP_8_R	GACTATCATCCGGGTGTGCTG
pCH-0112	0112-PrtPM_F	CTGGCTGTTATTTCATCTTACTC
pCH-0113	0113-PrtPM_R	GTTTACTGTAAGCATTTCAGAGG
pCH-0168	0168-pTLR_seq_F	CTATATCGTTAGGTACAGCTTCC
pCH-0170	0170-pTLR_seq_R_2	CGTGGCCAATATGGACAACTT
pCH-0173	0173-PrtMP_F_XhoI (pLP712)	GGCTCGAGGGCTGTTATTTCATCTTACTCG
pCH-0174	0174-PrtPM_R_NcoI (pLP712)	GGCCATGGGAACCGTTTCTACTCAATGAAC
